# Hypoxia upregulates the expression of the *NDRG1 *gene leading to its overexpression in various human cancers

**DOI:** 10.1186/1471-2156-5-27

**Published:** 2004-09-02

**Authors:** Hakan Cangul

**Affiliations:** 1 Department of Medical Genetics, Uludag University School of Medicine, Gorukle, Bursa 16059 Turkey; 2 Department of Environmental Medicine, New York University School of Medicine, New York, NY 10016 USA

## Abstract

**Background:**

The expression of *NDRG1 *gene is induced by nickel, a transition metal sharing similar physical properties to cobalt. Nickel may create hypoxia-like conditions in cells and induce hypoxia-responsive genes, as does cobalt. Therefore *NDRG1 *is likely to be another gene induced by hypoxia. HIF-1 is a transcription factor which has a major role in the regulation of hypoxia-responsive genes, and thus it could be involved in the transcriptional regulation of *NDRG1 *gene. Hypoxia is such a common feature of solid tumours that it is of interest to investigate the expression of Ndrg1 protein in human cancers.

**Results:**

Hypoxia and its mimetics induce in vitro expression of *NDRG1 *gene and cause the accumulation of Ndrg1 protein. Protein levels remain high even after cells revert to normoxia. Although HIF-1 is involved in the regulation of *NDRG1*, long term hypoxia induces the gene to some extent in HIF-1 knock-out cells. In the majority of human tissues studied, Ndrg1 protein is overexpressed in cancers compared to normal tissues and also reflects tumour hypoxia better than HIF-1 protein.

**Conclusions:**

Hypoxia is an inducer of the *NDRG1 *gene, and nickel probably causes the induction of the gene by interacting with the oxygen sensory pathway. Hypoxic induction of *NDRG1 *is mostly dependent on the HIF-1 transcription factor, but HIF-1 independent pathways are also involved in the regulation of the gene during chronic hypoxia. The determination of Ndrg1 protein levels in cancers may aid the diagnosis of the disease.

## Background

Cancer is a leading cause of death in humans, and current studies in the clinical area focus on either the early detection of this disease or on the development of new selective treatment tools. New tumour markers can provide aid in cancer diagnosis and create novel treatment opportunities.

Ndrg1, also named Cap43, Drg 1, RTP and rit42 following the discovery of its gene (*NDRG1*) in different laboratories, is a stress responsive protein which shuttles between cytoplasm and nucleus upon certain insults [1-5]. After its independent discovery in our lab [1], we worked on the expression of this gene and the availability of its protein product in human cells and tissues under different conditions.

Since our origin for the induction of the *NDRG1 *gene was nickel exposure, we worked on possible ways by which nickel could change the expression of this gene. Among the mechanisms investigated were epigenetic changes (DNA methylation and histone acetylation), signal transduction pathways (tyrosine phosphorylation, adenylate cyclase cascade, calmodulin, PKC, PI3-K), and ROS-mediated activation. However, none of these mechanisms were found to be involved in the induction of *NDRG1 *gene by nickel compounds [1,6-9].

On the other hand, it was shown that nickel induced the expressions of several hypoxia-responsive genes including vascular endothelial growth factor [10], erythropoietin [11], and glyceraldehyde-3-phosphate dehydrogenase [12]. Moreover, a well known hypoxia-mimicking agent is cobalt which is another transition metal adjacent to nickel in the periodic table. These facts led to the idea that the *NDRG1 *could be another hypoxia-responsive gene, and nickel could induce the expression of this gene creating hypoxia-like state in cells, as does cobalt. In the present study, the effects of hypoxia and hypoxia-mimicking agents on the *NDRG1 *gene expression have been investigated. Because HIF-1 transcription factor is a major regulator of hypoxia-responsive genes [13,14], the relationship between *NDRG1 *gene expression and HIF-1 has also been studied. Here, it is reported that hypoxia is an inducer of the *NDRG1 *gene, and that HIF-1 transcription factor is involved in the regulation of this gene, but HIF-1 independent pathways also exist in the induction of the gene in the case of chronic hypoxia.

The process of tumour expansion is characterized by rapid growth of cancer cells as the tumour establishes itself in the host. Accompanying this rapid growth are alterations in the cancer cell microenvironment, typically caused by an inability of local vasculature to supply enough oxygen and nutrients to the rapidly dividing tumour cells. This makes hypoxia one common feature of solid tumours [15]. Exploration of Ndrg1 protein expression patterns in various tissues showed that Ndrg1 protein was overexpressed in cancers compared to normal tissues. Because of its differential expression in cancer tissues and the high stability of the protein, Ndrg1 is proposed as a useful new tumour marker.

## Results

### Hypoxia and its mimetics induce in vitro expression of *NDRG1 *gene

**Figure 1:**
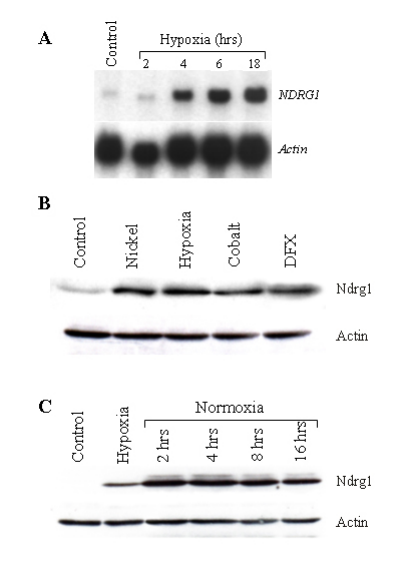
**The induction of *NDRG1 *gene expression by hypoxia and its mimetics. A) **A549 cells were exposed to normoxia (20% O_2_, control) for 20 hours or to hypoxia (0.5% O_2_) for the time periods indicated in the figure. 15 μg of total RNA was isolated and subjected to a Northern blot analysis as described in 'Methods' section. The blot first hybridized with *NDRG1 *probe (top panel), and then the membrane was stripped and rehybridized with *actin *probe (bottom panel) to show loading. **B) **A549 cells were exposed to 0.5 mM NiCl_2 _(Nickel), 200 μM CoCl_2 _(Cobalt), 200 μM desferrioxamine (DFX), or hypoxia (0.5% O_2_) for 20 hrs. 40 μg of whole cell protein extracts were loaded into each lane and subjected to Western blot analysis as described in 'Methods' section, using antibody against Ndrg1. Bottom panel (actin) shows loading. **C) **A549 cells were first exposed to hypoxia (0.5% O_2_) for 20 hrs, then taken out of hypoxic chamber and incubated additionally for the time periods indicated under normoxic (20% O_2_) conditions. Western blot analysis with antibody against Ndrg1 was carried out in whole cell protein extracts. Bottom panel (actin) shows loading.

**Figure 2:**
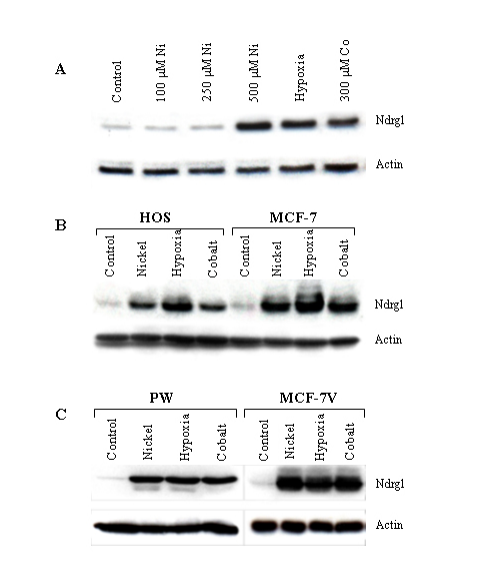
**The confirmation of Ndrg1 protein induction in different cell lines. (A) **HTE cells were incubated with different concentrations of NiCl_2 _(Ni), hypoxia, and 300 μM of CoCl_2 _(Co) for 20 hrs. **(B) **HOS and MCF-7, **(C) **PW and DU-145 cells were incubated with 500 μM of NiCl_2 _(nickel), hypoxia, and 300 μM of CoCl_2 _(cobalt) for 20 hrs. Western blot analyses were done as described in 'Methods' section. The membranes were first incubated with anti-Ndrg1 antibody (top panels), then stripped, and rehybridized with anti-actin antibody (bottom panels) to show loading.

To determine whether hypoxia could induce the transcription of the *NDRG1 *gene, A549 cells were exposed to hypoxia (0.5% O_2_) for different time periods. The RNA transcript of *NDRG1 *started to appear after 4 hours of incubation and increased up to 18 hours (Fig. 1A). To explore if this induction is also translated into protein response, Ndrg1 protein levels were determined after the exposure of cells to hypoxia and its mimetics. Figure 1B shows the accumulation of the protein following the incubation of A549 cells with these agents. To determine about the longevity of Ndrg1, at the end of hypoxia exposure cells were incubated under normoxic conditions for different time periods, and the levels of the protein were assessed. The results of this experiment showed that even after returning back to normoxia Ndrg1 protein levels remained elevated for at least 16 hours, indicating the stable nature of the protein (Fig. 1C). The induction of the Ndrg1 protein by hypoxia and transition metals has also been shown in several other cell lines (Fig. 2), confirming that this induction is a general phenomenon rather than being a cell-specific one.

### The regulation of *NDRG1 *expression by HIF-1 transcription factor

**Figure 3:**
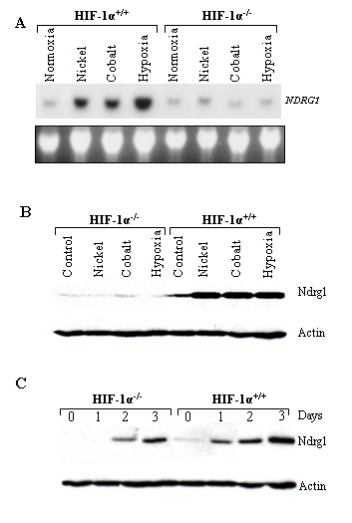
**The role of HIF-1 in the regulation of *NDRG1 *gene expression. A) **HIF-1 proficient (HIF-1α^+/+^) and deficient (HIF-1α^-/-^) cells were exposed to 0.5 mM NiCl_2_, 300 μM CoCl_2_, and hypoxia (0.5% O_2_) for 20 hrs. 15 μg of total RNA was isolated and subjected to a Northern blot analysis using *NDRG1 *probe (top panel). Ethidium bromide staining was used to adjust loading (bottom panel). **B) **HIF-1α^+/+ ^and HIF-1α^-/- ^cells were exposed to 0.5 mM NiCl_2_, 300 μM CoCl_2_, and hypoxia (0.5% O_2_) for 20 hrs. 40 μg of protein extracts were subjected to Western blot analysis using antibody against Ndrg1 protein (top panel). Actin bands in the bottom panel show loading. **C) **Cells were first incubated for 24 hours under normoxic conditions for attachment (Day 0) and then exposed to hypoxia for up to three days. Western blot analysis with antibody against Ndrg1 was carried out in 25 μg of whole cell protein extracts (top panel). Actin bands in the bottom panel show loading.

HIF-1 is a heterodimeric transcription factor consisting of HIF-1α and HIF-1β subunits. It is expressed by all cells of the human body in response to hypoxia and contributes to the regulation of hypoxia-responsive genes. HIF-1α is the unique, O_2_-regulated subunit that determines HIF-1 activity whereas HIF-1β is expressed ubiquitously and is a common partner of several other proteins. Therefore, the elimination of HIF-1α expression completely prevents the formation of HIF-1 protein. To investigate the relationship between HIF-1 transcription factor and the expression of *NDRG1 *gene, HIF-1 proficient (HIF-1α^+/+^) and deficient (HIF-1α^-/-^) cells were exploited. Short-term hypoxia (20 hrs) experiment showed that *NDRG1 *mRNA was induced in HIF-1 proficient but not in deficient cells (Fig. 3A). The transcriptional induction of the gene by both nickel and cobalt was also dependent upon HIF-1 transcription factor. These results have been confirmed with the data obtained at the protein level: the same agents (short-term hypoxia, nickel, and cobalt) caused the accumulation of Ndrg1 protein in HIF-1 proficient but not in deficient cells (Fig. 3B).

In another experiment, HIF-1α^+/+ ^and HIF-1α^-/-^cells were exposed to long-term hypoxia up to three days, to simulate the chronic conditions that cancer cells go through. The results revealed that hypoxia increased Ndrg1 protein levels even in HIF-1α^-/- ^cells starting from the second day of hypoxia (Fig. 3C). However, the level of protein accumulation on the third day was considerably higher in HIF-1α^+/+ ^cells than in HIF-1α^-/- ^cells. These observations implied that the Ndrg1 protein induction was not totally dependent on HIF-1 transcription factor, and that some other pathways were also involved in the induction of Ndrg1 protein by long-term hypoxia.

### The detection of Ndrg1 and HIF-1α protein levels in normal and cancerous human tissues

**Figure 4:**
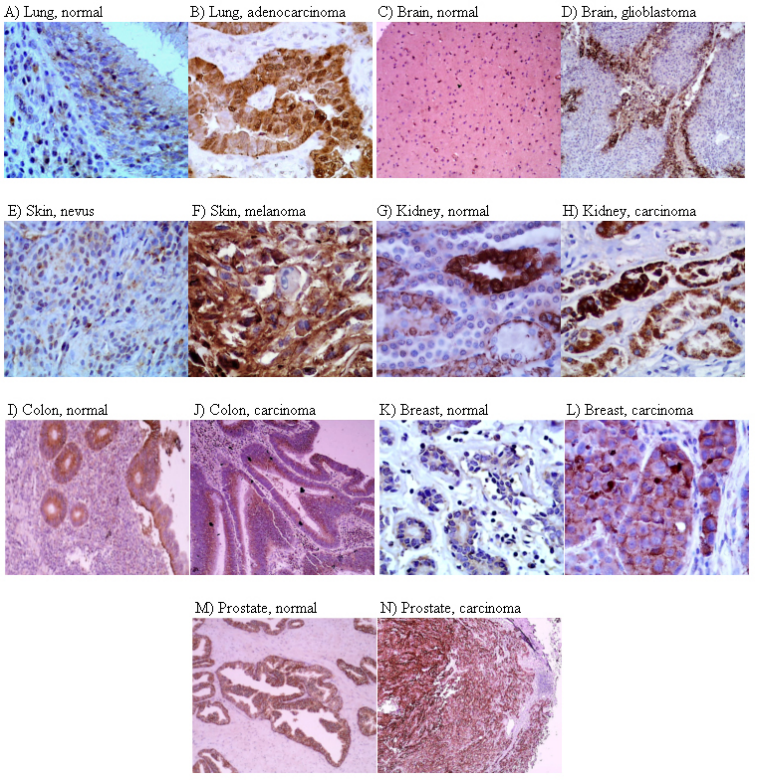
**Immunohistochemical detection of Ndrg1 protein in human tissues. **The nature of the tissue is indicated on top of each picture. Original magnifications are as follows: A, ×400; B, ×400; C, ×100; D, ×100; E, ×400; F, ×400; G, ×400; H, ×400; I, ×100; J, ×100; K, ×400; L, ×400; M, 100; N, ×100.

**Figure 5:**
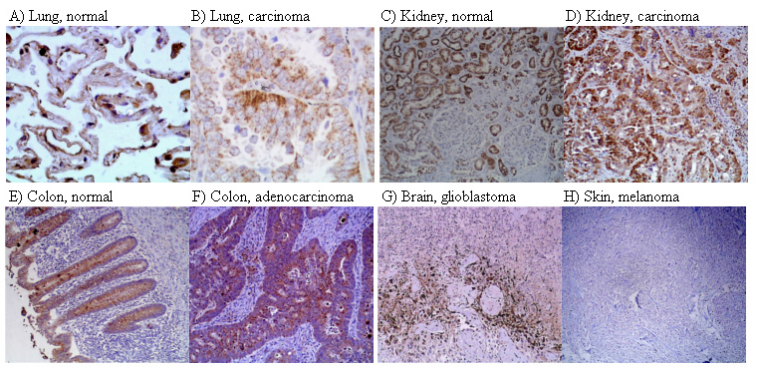
**Immunohistochemical detection of HIF-1α protein in human tissues. **The nature of the tissue is indicated on top of each figure. Original magnifications are as follows: A, ×400; B, ×400; C, ×100; D, ×100; E, ×100; F, ×100; G, ×40; H, ×40.

Figure 4 shows a variety of human normal and cancer tissues stained immunohistochemically with anti-Ndrg1 polyclonal antibody. To understand whether elevations of Ndrg1 protein coincided with the expression of HIF-1 transcription factor, we also stained the tissues with an antibody to HIF-1α (Fig. 5). In lung, Ndrg1 preferentially stained malignant cells including both non-small and small cell types, whereas surrounding normal tissue remained negative for staining (Fig. 4A,4B). In contrast to Ndrg1, HIF-1α was present in both normal and lung cancer cells at similar levels (Fig. 5A,5B, and Table 1). As shown in Figure 5B, HIF-1α was present at higher levels in some cancer cells but not to the extent of Ndrg1 (Fig. 4B). In brain tissue, Ndrg1 antibody selectively stained cancer cells whereas normal brain remained negative for this staining. Figure 4C shows the normal brain tissue staining and Figure 4D shows human glioblastoma multiforme. Ndrg1 preferentially stained the tumour cells adjacent to the necrotic areas which were supposed to be hypoxic. In both astrocytomas and hemangioblastomas there was intense staining for both Ndrg1 and HIF-1α in a number of different patients. Skin cancer melanoma cells showed the most intensive staining with Ndrg1 antibody (Fig. 4F), and a benign skin lesion nevus had very limited Ndrg1 staining (Fig. 4E). In contrast, staining with HIF-1α antibody showed little effect in melanoma cells (Fig. 5H). Ndrg1 protein was generally found at low levels in most normal tissues with the exception of some higher expression in the distal and proximal convoluted tubule of the kidney (Fig 4G, and Table 1). The distal and proximal convoluted tubules of the kidney also expressed HIF-1α (Fig. 5C). There was also expression of Ndrg1 protein in normal colon mucosa and smooth muscle, as well as some expression in normal breast and prostate (Fig. 4I,4K, and 4M). However, with the exception of the colon samples, the expression of Ndrg1 protein in cancer cells of these tissues was considerably higher (Fig. 4L,4N). In normal human tissues that showed immune reactivity to Ndrg1 antibody (such as kidney, prostate, breast, and colon) staining was emphasized particularly in glandular structures and tubular epithelia. Table 1 summarizes the total number of tissues and staining intensities. As seen from the table, differential expression between normal and cancer tissues was much more apparent in Ndrg1 than HIF-1α.

**Table 1:** The presence of Ndrg1 and HIF-1α proteins in various normal and malignant human tissues

**TISSUE (n)**	**Ndrg1**				**HIF-1α**			
	-	+	++	+++	-	+	++	+++
Normal Lung (30)	24	6	-	-	4	11	15	-
Lung Cancer (30)	-	-	3	27	-	6	21	3
Normal Liver (20)	7	13	-	-	2	15	3	-
Liver Cancer (20)	-	-	4	16	-	11	2	7
Normal Breast (18)	3	13	2	-	1	9	8	-
Breast Cancer (20)	-	-	6	14	-	4	7	9
Smooth Muscle (30)	12	15	3	-	9	11	10	-
S.M. Cancer (24)	-	6	7	11	-	9	12	3
Normal Brain (28)	24	4	-	-	18	10	-	-
Brain Cancer (36)	-	-	11	25	-	-	15	21
Normal Kidney (15)	-	6	8	1	-	5	10	-
Kidney Cancer (22)	-	-	7	15	-	7	9	6
Normal Skin (20)	14	6	-	-	11	9	-	-
Melanoma (10)	-	-	-	10	4	6	-	-

Tissues were stained immunohistochemically with antibodies against Ndrg1 and HIF-1α as described in 'Methods' section. Each value shown in the table is from a single tissue sample from an individual patient. The numbers in parentheses (n) represent the total numbers of tissues stained with both antibodies. The expression levels were marked as follows: (-) no staining; (+) weak; (++) moderate; (+++) overexpression.

## Discussion

Experiments conducted in this study provide clear evidence that hypoxia and its mimetics induce the expression of *NDRG1 *gene at both the RNA and protein level (Figs. 1, 2, and 3). It is hypothesized that nickel induces this gene by creating hypoxia-like conditions in cells. Support for this hypothesis came with the discovery of the first molecular oxygen sensors in mammalian cells, namely proline and asparagine hydroxylase enzymes which regulate the oxygen-dependent post-translational modification of HIF-1α protein and thereby change its stability and transcriptional activity. Prolyl hydroxylase PHD hydroxylates HIF-1α at residue Pro^564 ^in the presence of oxygen which creates a signal for pVHL to bind it, causing consecutive ubiquitination and proteasomal degradation of HIF-1α protein [16,17]. Likewise, in the presence of oxygen asparaginyl hydroxylase enzyme FIH-1 (factor inhibiting HIF-1) hydroxylates C-TAD domain of HIF-1α which in turn prevents it binding to coactivator p300/CBP and limits transactivation ability [18-21]. Under hypoxic conditions these modifications of HIF-1α by above mentioned enzymes do not occur, and the transcription of hypoxia-responsive genes are promoted [22-24].

The hypoxia-responsive element (HRE) is a specific five nucleotide-HIF binding-DNA sequence (5'-RCTCG-3') which is common in all hypoxia-responsive genes [25,26]. *NDRG-1 *gene has three HIF-1 binding sites in its non-coding sequence, one in its promoter and the other two in the 3' untranslated region [27]. It is known that a HIF-1 binding site in the 3' region of the erythropoietin gene regulates the transcription of this hypoxia-responsive gene [28]. Conceivably, *NDRG1 *is likely to be regulated by HIF-1 through the binding sites in its untranslated sequences.

HIF-1 modifier enzymes, PHD and FIH-1, both have non-heme iron centres (29), and transition metals nickel and cobalt can interact with these centres, subsequently inhibiting the enzymes (30). By their effects on HIF-1 modifying enzymes, nickel and cobalt have the capacity of creating constitutive hypoxia-like conditions in cells [31-33].

**Figure 6:**
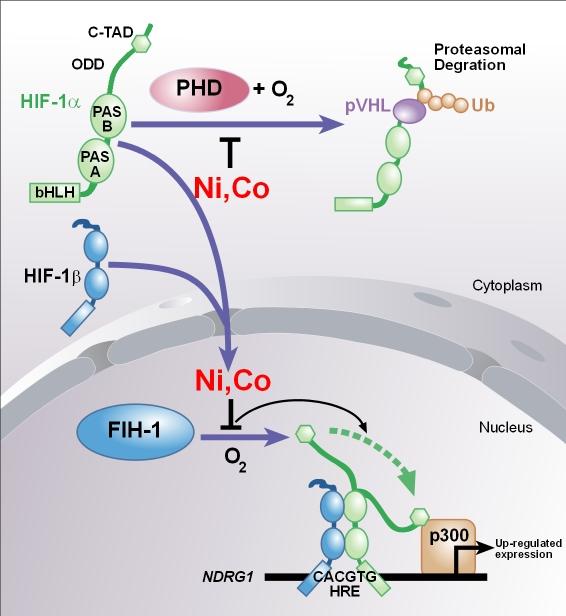
The illustration of the hypothetical mechanism by which nickel and cobalt upregulate the expression of the *NDRG1 *gene

Our hypothesis relating nickel, oxygen-sensing, hypoxia, HIF-1, pVHL, and *NDRG1 *expression may be elaborated as follows: when we expose cells to nickel, internalized nickel inhibits PHD enzyme interacting with its iron centre. This prevents the hydroxylation of proline residue in the ODD domain of HIF-1α and subsequent pVHL binding, rescuing HIF-1α from proteasomal degradation (Fig. 6, *upper part*). Rescued and accumulated α subunits of HIF-1 form stable heterodimers with β subunits and translocate to the nucleus, and HIF-1αβ heterodimers bind to hypoxia responsive elements (HRE) of the *NDRG1 *gene and promote the transcription of the gene. Nickel also inhibits the FIH-1 enzyme and subsequent hydroxylation of C-TAD domain of HIF-1α, and this in turn results in the recruitment of coactivators of HIF-1 to the *NDRG1 *gene regulatory sequences thereby further stimulating the expression of the gene (Fig. 6, *lower part*). However, since the experiments to show the specific interaction between metals and HIF-1 modifying enzymes are yet to be executed, this aspect of the hypothesis remains unproven.

Ndrg1 protein outlasts HIF-1 after hypoxia. Despite being a major regulator of hypoxia response, HIF-1 transcription factor is a very unstable protein which is rapidly degraded under normoxic conditions; the half-life of HIF-1α in post-hypoxic cells is less than 5 minutes. On the other hand our results showed that Ndrg1 protein levels remain high at least 16 hours after returning to normoxic conditions (Fig. 1C). Similar results have been reported by Lachat et al. (34) who showed that it took 48 hours for Ndrg1 levels to return to pre-anoxic levels after the cessation of hypoxia. Their experiment carried out in colon carcinoma cells (SW480) supports our results, indicating that the high stability of Ndrg1 protein is not cell specific.

Our study of relationship between HIF-1 and *NDRG1 *expression indicated that the induction of the gene was primarily dependent on this transcription factor (Fig. 3). Neither RNA nor the protein product of *NDRG1 *gene was induced in HIF-1α^-/- ^cells upon short-term exposures to hypoxia. In the long-term hypoxia experiment we detected some amount of Ndrg1 protein in HIF-1α^-/- ^cells starting from the second day, but the levels of protein accumulation on the second and third days were considerably higher in HIF-1α^+/+^cells than those in HIF-1α^-/- ^cells. However, these results indicate that in chronic hypoxic conditions such as cancer, other factors additional to HIF-1 could be involved in the regulation of *NDRG1 *gene expression. Several HIF-1 independent pathways have been described to date as being effective under hypoxic conditions (35–40).

Our work in several human tissues showed that in the majority of these organs Ndrg1 protein was differentially overexpressed in cancers compared to normal tissues (Fig. 4, Table 1). Normal tissue samples of certain organs (lung and brain) were almost completely free of Ndrg1 expression, whereas these samples showed HIF-1 protein expression to some extent. In some cases (especially glioblastoma of brain) the expression of Ndrg1 coincided with HIF-1 protein, indicating that induction of HIF-1α by hypoxia probably resulted in Ndrg1 accumulation in these cancers. In most of the other cases though, diffuse and strong Ndrg1 expression did not coincide with HIF-1 protein expression. These differences in the detection of two proteins may be explained by (i) considerably higher stability of Ndrg1 protein compared to that of HIF-1, and (ii) reflection of HIF-1 independent hypoxia response by Ndrg1. With these features, Ndrg1 has the capacity of reflecting tumour hypoxia in a broader spectrum than does HIF-1 and could be considered as a better signature for hypoxic tumour cells than HIF-1. Therefore, despite the proposal of HIF-1 as a tumour marker [41], we present its down-stream product Ndrg1 as a stronger candidate of cancer marker especially for certain tissues (lung, brain, and skin).

Several normal tissue samples showed some Ndrg1 expression albeit at lower levels than in cancer samples of similar tissues. In their comprehensive study, Lachat et al. (34) showed the expression of Ndrg1 protein in normal human tissues, reporting also the intensities and sub-cellular localizations of the stainings. We observed similar staining patterns in several tissues; more emphasized stainings in the glandular, acinar, ductal, and tubular cells of normal breast, prostate, colon, and kidney tissues. We also share the observation that Ndrg1 exist in all three locations of the cells-cytoplasm, nucleus, and membrane. But, with the exception of colon mucosa, when we stained the cancerous tissues of above mentioned organs, the staining was more intense. However, since the differential expression of Ndrg1 between normal and cancer tissues of lung, brain, and skin was much starker (Table 1), we propose Ndrg1 be initially tried as a marker for these tissues. For the reasons that are unknown, Ndrg1 was expressed at lower levels in colon cancer than it was in normal colon. Similar results have also been reported by others [42]. This could be due to fact that colon epithelium is a dynamic structure being continuously renewed. Studies addressing the mechanism of Ndrg1 down-regulation in colon cancers will shed more light on the function of Ndrg1 protein and its relation to cancer development.

Another common finding between our study and Lachat et al's (34) is no expression of Ndrg1 in normal brain and lung epithelium. Lachat et al. (34) showed at the transcriptional level that normal brain and lung express *NDRG1*, in fact these and many other tissues expressing *NDRG1 *mRNA did not contain detectable levels of the protein product of this gene. This could be due to degradation of the mRNA under normal conditions. Stabilizing the mRNAs of hypoxia-responsive genes is one way cells promote the expression of these genes under hypoxic conditions (38, 39). More studies are needed to resolve how Ndrg1 levels are managed in normal cells, during hypoxia, and as well as in cancer cells.

Masuda et al. [40] report the down-regulation of *NDRG1 *gene by VHL tumour suppressor protein (pVHL). They state that no hypoxia-responsive element exists on the 5' flanking sequence of the gene and thus underplay the role of HIF-1 in the regulation of *NDRG1*. As mentioned previously, *NDRG-1 *gene has three HIF-1 binding sites, one in its promoter and two in the 3' untranslated region. Therefore, the down-regulation of *NDRG1 *gene by pVHL is likely to be mediated through the HIF-1 pathway. The observation of *NDRG1 *being down-regulated by a major tumour suppressor further supports our observation that it is up-regulated in several cancer tissues and could be used as a marker.

Hypoxia-responsive pathway (HRP) allows tumour cells to overcome harsh microenvironment conditions associated with tumour growth. The protein products of induced by this pathway (e.g. EPO, VEGF, several glycolytic enzymes) allow clones of tumour cells to gain growth advantage under unfavourable conditions, and this concept is pivotal in switching to a more malignant phenotype. Although the exact functions of Ndrg1 are still unknown, as another effector of HRP, it is also likely to help tumour cells establish themselves. Therefore, the use of drugs that specifically disrupt the functions of Ndrg1 protein may provide new cancer therapies. It is thus of interest to investigate the effects of the elimination of this protein on the cancer cell survival and proliferation. However, first Ndrg1 expression in normal hypoxic tissue (such as infarct tissues) should be determined to show the cancer specificity of the protein. Second, the potential side effects of its elimination should be assessed since several normal tissues express Ndrg1 ubiquitously.

Hypoxia is also an important determinant for the success of chemotherapy and radiotherapy [43]. Masuda et al. [40] even argue that Ndrg1 could be involved in limiting sensitivity to anti-cancer drugs. However hypoxia is an equally likely limiting factor in anti-cancer therapy, and Ndrg1 may be simply the signature of the hypoxic state.

## Conclusions

Hypoxia induces the *NDRG1 *gene, and nickel probably causes the induction of the gene by interacting with the oxygen sensory pathway. Hypoxic induction of *NDRG1 *is mostly dependent on HIF-1 transcription factor. However, regulation of the gene in long term hypoxia involves some other HIF-1 independent pathways.

Ndrg1 protein was overexpressed in the majority of cancers studied here. With the exception of colon cancer, staining with Ndrg1 antibody distinguished between normal and tumour cells in most cancerous tissues. The mechanism of this overexpression is related to the hypoxic state of cancer cells. Ndrg1 protein is a better indicator of tumour hypoxia than HIF-1 in immunohistochemical analyses. This is probably due to the stable nature of the Ndrg1 protein compared to the very unstable HIF-1 protein, and also to capacity of Ndrg1 in reflecting HIF-1 independent hypoxia response. Therefore, the determination of Ndrg1 protein in tissue samples may provide a more useful tool for cancer diagnosis. Even though the exact functions of Ndrg1 protein are still unknown, as another effector in hypoxia-response, it can help cancer cells to survive and grow under unfavourable conditions. Therefore, it may also be possible to direct therapy towards Ndrg1 protein using drugs that specifically disrupt the functions of this protein.

## Methods

### Cell lines and culture conditions

Human lung cancer cell line A549 (CCL185), human osteosarcoma cell line HOS (CRL 1543), human mammary carcinoma cell line MCF-7 (HTB 22), and human prostate cancer cell line DU-145 (HTB 81) were purchased from American Type Culture Collection (Rockville, MD, USA). Human trachea epithelium (HTE) cells, HIF-1α^+/+^, and HIF-1α^-/- ^fibroblasts were gifts from Dr. Konstantin Salnikow of NYU. The production of HIF-1α^+/+ ^and HIF-1α^-/- ^fibroblasts was described elsewhere [44]. PW cells were a gift from Dr. Qunwei Zhang of NYU.

The cell lines were maintained at 37°C as monolayers in a humidified atmosphere containing 5% CO_2_. Cells were passaged by trypsinization when they reached 70–80% confluence. A549 cells were grown in Ham's F-12K nutrient mixture (Kaighn's modification) supplemented with 10% fetal bovine serum (FBS) and 1% penicillin/streptomycin [equivalent to 100 units (U)/ml and 100 μg/ml, respectively]. MCF-7, PW, HIF-1α^+/+^, and HIF-1α^-/- ^cells were maintained in DMEM (Dulbecco's modified Eagle's medium) with the same supplements. HOS and HTE cells were grown in α-MEM (α-minimal essential medium) additionally supplemented with 2 mM L-glutamine. For Northern and Western blot experiments, 5 × 10^5 ^cells in 10 ml of media were plated in each of 10-cm dishes (Corning Inc, Corning, NY). Cell numbers were determined using the ZM Coulter Counter (Coulter Electronics, England). To render cells hypoxic, dishes were placed in an incubator chamber flushed with 95% N_2 _and 5% CO_2_. This resulted in approximately 0.1–0.5% O_2 _after several hours. After 20 hours, cells were released from hypoxia and quickly scraped in ice-cold phosphate-buffered saline (PBS), and analyses were performed as described below.

### Northern blot analysis

Total RNA was extracted from cells immediately after exposures by using TRIzol reagent (Gibco-BRL) according to instructions of the manufacturer. 15 μg of RNA/lane was separated by electrophoresis in 1.0% agarose-formaldehyde gels and then transferred to nitrocellulose membranes (BA-85; Scleicher & Schuell). *NDRG1 *and *actin *probes were labelled with [α-^32^P]dCTP by using a randomly primed-DNA labelling kit (Promega). Cloning of *NDRG1 *gene was as described previously [1]. The blot first hybridized with *NDRG1 *probe, and then the membrane was stripped and rehybridized with *actin *probe to show loading. The bands were visualized by exposing X-ray films (Eastman Kodak Co, NY, USA) to hybridized membranes.

### Western blot analysis

Cells were lysed, and proteins were harvested in 125 μl of TNES buffer [50 mM Tris-HCl (pH: 7.5), 2 mM EDTA, 100 mM NaCl, 1 mM sodium orthovanadate (Na_3_VO_4_), 10 mM sodium fluoride, and 1% NP40] containing protease inhibitors (PMSF 1 mM, aprotinin 1 μg/ml, leupeptin 5 μg/ml, and chymostatin 2 μg/ml). 40 μg or 25 μg of protein was loaded into each lane of a 10% SDS-PAGE gel and separated by electrophoresis. Proteins were then transferred to PVDF membranes (Roche Diagnostics Co, IN, USA), and the membranes were first incubated with rabbit anti-Ndrg1 polyclonal antibody at the dilution of 1:1000 in 5% non-fat dry milk for one hour at room temperature. The antibody production is described elsewhere [3]. After washing four times for 15 min each with TBS buffer, the membranes were incubated with a second anti-rabbit peroxidase-conjugated antibody (Santa Cruz, CA, USA) at the dilution of 1:10^4 ^in 5% milk for one hour at room temperature. At the end the membranes were treated with chemiluminiscent substrate ECL (Amersham Pharmacia Biotech, UK) for 1 min at room temperature, and Biomax MR-1 films (Eastman Kodak Co, NY, USA) were exposed to the membranes. The molecular weight of Ndrg1 was determined using prestained molecular weight markers (Invitrogen Life Technologies, CA, USA).

### Tissue staining

For in vivo detection of Ndrg1 protein in human cancer and normal tissues, immunohistochemical (IHC) staining was exploited, using a rabbit polyclonal antibody against a 30-aminoacid-sequence at the C-terminal end of the Ndrg1 protein [3]. Tumour and normal tissue sections were obtained from the tumour registry of the Cancer Institute of New York University Medical School. The tissues were embedded in paraffin wax. Five-micron sections were cut and baked at 60°C for 30 minutes. After cooling, the sections were deparaffinized and hydrated through the following series: 3 × 5 minutes xylene, 3 × 5 minutes 100% Etoh (ethyl alcohol), 3 × 5 minutes 95% Etoh. The slides were then rinsed gently with distilled water and stained with hematoxylin-eosin for histopathological diagnosis. For antigen retrieval, the slides were heated in 1 mM EDTA buffer (pH: 8.0) in a microwave oven for 10 min, and then endogenous peroxide was blocked with methanol containing 0.35% H_2_O_2 _for a further 30 min. After incubation with the antibody against Ndrg1 protein overnight, the slides were processed with a second anti-rabbit peroxidase-conjugated antibody (Santa Cruz, CA, USA). Identification of the protein was then achieved using Avidin-Biotin horseradish peroxidase complex and 3,3-diaminobenzidine (DAB) as the chromogen. Negative controls were performed using nonimmune serum instead of primary antibodies.

Immunohistochemical detection of HIF-1α protein was achieved by using Catalyzed Signal Amplification System (DAKO Corp., Carpinteria, CA) which is based on streptavidin-biotin-horseradish peroxidase complex formation. Antigen retrieval was done by 10 mM sodium citrate buffer (pH 6.0). The specimens were incubated overnight at +4°C with monoclonal anti-HIF-1α antibody (Clone MAb H1α 67, #NB 100–123; Novus Biologicals, Littleton, CO) in a dilution of 1:1000. After the amplification of the signal according to manufacturer's instructions, the slides stained with chromogen DAB, counter-stained with hematoxylin, dehydrated, and then mounted.
